# Where Rabies Is Not a Disease. Bridging Healthworlds to Improve Mutual Understanding and Prevention of Rabies

**DOI:** 10.3389/fvets.2022.867266

**Published:** 2022-06-15

**Authors:** Deborah Nadal, Katie Hampson, Tiziana Lembo, Rebecca Rodrigues, Abi Tamim Vanak, Sarah Cleaveland

**Affiliations:** ^1^Institute of Biodiversity, Animal Health & Comparative Medicine, College of Medical, Veterinary & Life Sciences, University of Glasgow, Glasgow, United Kingdom; ^2^Center for One Health Research, School of Public Health, University of Washington, Seattle, WA, United States; ^3^Centre for Biodiversity and Conservation, Ashoka Trust for Research in Ecology and The Environment, Bangalore, India; ^4^School of Life Sciences, University of KwaZulu-Natal, Westville, South Africa; ^5^DBT/Wellcome Trust India Alliance Program, Hyderabad, India

**Keywords:** dog bites, dog-mediated rabies, human rabies, faith healing, healthworlds, mass dog vaccination, One Health, post-exposure prophylaxis

## Abstract

Deeply embedded in local social, cultural, and religious settings, traditional healing is part of dog bite and rabies management in many rabies endemic countries. Faith healing, which usually encompasses a more holistic approach to health including physical, mental and social dimensions, is rare in the context of rabies. In Gujarat, Western India, the Hindu goddess Hadkai Mata is worshiped by low-caste communities as the Mother of Rabies in the event of a dog bite to a person or their livestock. This belief might influence people's attitudes and behaviors toward rabies prevention but has never been investigated. Through 31 in-depth interviews with healers and staff of Hadkai Mata temples, this paper explores the system of knowledge around dog and human rabies that is built and shared in these places of worship and healing. Qualitative and quantitative data were analyzed looking for convergences and divergences with the recently launched National Action Plan for dog-mediated Rabies Elimination. Results suggest that while the etiology of human rabies as a social illness is usually explained as the goddess's wish to correct misbehaving people and restore positive interpersonal relations, there is some appreciation for the biological processes of infection that lead to rabies as a physical disease. Hadkai Mata is believed to cure rabies if her patients undergo the necessary process of moral growth. Although conventional post-exposure prophylaxis is not opposed *per se*, it is often delayed by patients who seek traditional treatment first. Some reluctance was expressed toward mass dog vaccination because it is seen as an interference in how the goddess controls dogs, by enraging them—hence infecting them with rabies—and sending them to bite wrongdoers. Addressing these cultural perceptions is likely to be critical in achieving effective control of dog rabies in this region. The study highlights the value of multidisciplinary approaches in the control and elimination of rabies, as well as other zoonoses. This includes the importance of understanding different culturally- and religiously- mediated ways in which humans relate to animals; and looking for points of convergence and mutual understanding, upon which context-tailored, linguistically-accurate, locally acceptable, feasible and effective strategies can be designed.

## Introduction

Rabies constitutes an important public health concern and causes an estimated 59,000 human deaths, with the highest burden falling on the rural poor of Asia and Africa (1). This viral infection is usually transmitted through animal bites, mostly from domestic dogs, and is fatal once clinical signs appear (2). Based on the One Health concept that recognizes the interdependence between human and animal health, the global strategic plan to end human deaths from dog-mediated rabies by 2030 (“Zero by 30”) consists of vaccinating dogs to stop transmission at its source, providing post-exposure prophylaxis (PEP, which uses rabies vaccine and, in case of severe exposure, rabies immunoglobulin) to exposed individuals, and increasing community awareness about these two measures and dog bite prevention generally (3). In most endemic countries, each of these components faces challenges. The design of mass dog vaccination campaigns is often poorly informed by an understanding of people's attitudes toward dogs and perceptions around dog vaccination (4); PEP delivery struggles to reach those that need it most (5); and awareness about how to manage dog bites is often low not only amongst the public (6) but also healthcare providers (7). The common denominator of these issues is frequently a disconnect between research and policy (8), hence the design of strategies that are not context-specific (9).

India shares one-third of the global human rabies burden (10) and typifies these problems. Despite the high burden of disease, rabies was made notifiable only in September 2021 (11) and therefore its on-the-ground reality is still poorly understood. Inspired by “Zero by 30,” India recently launched its National Action Plan for dog-mediated Rabies Elimination (NAPRE). Enthusiastic about this plan, a Union Minister claimed that “*The mere mention of hadakwa* [“rabies” in the language of his native State, Gujarat] *induces terror in rural areas. They* [villagers] *will actively help the government in this noble endeavor”* (12).

Based on worldviews, beliefs, and symbols, faith healing consists of religious and spiritual practices, performed by the patient or an intermediary, to alleviate suffering or restore health. Health is usually understood not only as the absence of disease, but more specifically as the co-presence of physical, mental, and social wellbeing, and can be achieved in several ways (for example, through divine energy, the power of healer's hands, a process of spiritual growth). In relation to rabies and dog bites, faith healing is usually considered part of traditional medicine, together with the more common, both in India and abroad, application of substances on the wound (13, 14). India also has the unique distinction of having, besides biomedicine, six other official systems of medicine (i.e., Ayurveda, Siddha, Unani, Yoga, Naturopathy, and Homeopathy), the first three of which are used in the field of rabies (15). For example, a previous study found evidence for herbal therapy and “magico-religious [practices]” being sought by rabies bite victims in 60% of fatal cases in India (10).

By influencing individual and community dog keeping practices and concepts of animal welfare, religion also impacts mass dog vaccination, but this aspect of rabies control is rarely studied (16), even by anthropologists (17). For example, people in India feed free-roaming dogs more because of the belief in karma (i.e., good deeds in the current life lead to a happier rebirth) than a genuine concern for dog welfare (4).

Here we explore the role played by the Hindu goddess Hadkai Mata (literally, the Mother of Rabies) in the understanding of and approach to dog and human rabies in Gujarat, the only Indian State where the goddess is venerated. To our knowledge, Hadkai Mata is, among the world's current religions, one of the very few rabies-related deities who inspire the performance of faith-healing practices. In the past, the figure of Lyssa in Greek mythology and the Christian saints Quiteria and Denis were associated with rabies, but not with a specific treatment. Saint Hubert was a notable exception. Until the early twentieth century, people in Europe hung metal nails known as the keys of Saint Hubert on the walls of their houses to protect themselves from rabies. In case of a bite, the key was heated and used to cauterize the wound, and a thread—supposedly from the saint's stole—was inserted in a little incision on the patient's forehead for 9 days, during which time dietary restrictions were followed (18).

Theoretically, this paper reflects on two very different approaches to the understanding of people's behaviors; the extended parallel processing model and the concept of “healthworlds.” Within health behavior change theories, the extended parallel processing model states that, for people to take protective action against a health threat and eventually improve community participation in disease control programs, health communication campaigns should focus on two factors. They are people's fear—or “terror,” to echo the abovementioned Union Minister's words—and perceived self-efficacy (i.e., a person's confidence in their capacity to put a proposed solution into practice) (19). Despite the lively debate on whether interventions based on behavioral theory really work in the real world (20), with rabies, fear appears to be a potent driver of community engagement and compliance with human and dog vaccination, for example in both Tanzania (21) and Peru (22). In this paper, we challenge this assumption in two ways. First, by highlighting the importance of understanding rabies dynamics in local, context-specific variations. Second, by acknowledging that behaviors are not the mere result of individual choices and cognitive determinants (23), but they reflect people's healthworld. Crucial to understanding “the empirical complexity of health beliefs (importantly, including religion) and behaviors” (24), healthworlds include large-scale social and economic relationships. They are concerned with disease (i.e., the pathological deviation from a biological norm) and illness (i.e., the patient's experience of ill health). They also adopt expanded definitions of medical pluralism [i.e., “shopping and switching” between multiple modalities of care available, in a complementary or alternative way—(25)] that include faith-based options. According to this concept, which resonates with systems-based approaches to health like EcoHealth and One Health (26), “individuals' healthworlds are shaped by, and simultaneously affect, the socially shared healthworld constituted by the collective search for health and wellbeing” (24). What inspired the development of this concept, and its use in this paper, is the ultimate, practical purpose, *via* communicative action (27), of having different people seek to reach an understanding about something so that they can coordinate their plans of action by way of mutual agreement.

No religious or devotional literature exists on Hadkai Mata, because this deity exists only in the oral tradition. Scholarly research is extremely limited (28, 29) and the relation between Hadkai Mata and rabies management has never been investigated. In this paper, based on discussions with traditional healthcare providers at Hadkai Mata temples, we analyze some aspects of the cult of this goddess to understand whether and how it influences the way this deity's followers manage dog bites and rabies. In particular, we ask the following questions: (1) what knowledge on rabies is collectively built and/or circulated in Hadkai Mata temples?; (2) what do this deity's believers do when they are bitten by a dog, and why?; and (3) what is the opinion of the traditional healthcare providers about dog vaccination (which has never been done at scale in rural India before), and why? The ultimate purpose of this work is to identify convergences and divergences (30) between this local perspective on rabies and the recently launched NAPRE. This understanding may enable us to propose tailored, bottom-up recommendations that address the wellrecognized limitations of top-down public health interventions that can be disconnected from local realities. Anthropology and implementation research literature already abound with examples of wasted resources and ineffective interventions as a result of mistrust from communities, and aggravated social fractures over power imbalances and communicative injustice (31–34). Given the size, geographic variety, federal political system, cultural diversity, and socio-economic complexity of India, the risk of failure of a one-size-fits-all strategy is evidently high.

## Materials and Methods

### Study Area

Of the 36 States and Union Territories of India, Gujarat ranks fifth by area (196,024 sq km) and ninth by population [60,439,692 people, in 2011—(35)]. Agriculture and industry lead its economy, with the pharmaceutical sector playing a key role in the country's drug manufacturing. Gujarat ranks 10th by per capita gross state domestic product [3,200 USD, in 2019—(36)] and its 18,225 villages benefit from a good infrastructure, with eight villages out of 10 reachable through all-weather roads and nearly all of them connected to the electrical grid (37).

Nevertheless, 17% of Gujaratis live below the poverty line [20 USD per month, in 2012—(38)] and in 2019, the state ranked 21^st^ by human development index (39), mainly because of caste- and religion-based social inequality (40) and insufficient spending on social security, rural development, and education (41). Most Gujaratis are Hindus (89%) and live in rural areas (57%). The population is classified as 15% Scheduled Tribes (geographically, and often economically, isolated communities), 7% Scheduled Castes (groups that face caste-based ostracization), 51% Other Backward Castes (historically oppressed communities, who continue to face some social and educational isolation), and 27% Other Communities (upper-caste Hindus and most non-Hindus) (35).

The percentage of the State budget spent on healthcare (5%) is far below the 8% recommended by the National Health Policy, and the privatization of the health sector is on the rise, with public health infrastructure being primarily affected (42). With regards to PEP, unlike in most Indian States, Gujarat's government hospitals usually have good stocks of rabies vaccines (43), which should be provided free of cost at such facilities, as per a nationwide policy. Similarly, hospitals in the state provide human rabies immunoglobulin (HRIG) for free (44). In most of the country, HRIG, which is generally preferred by doctors to its cheaper, equine alternative, is usually available only in private clinics and for a fee that only high-income patients can afford.

### Study Sites

**Figure 1:**
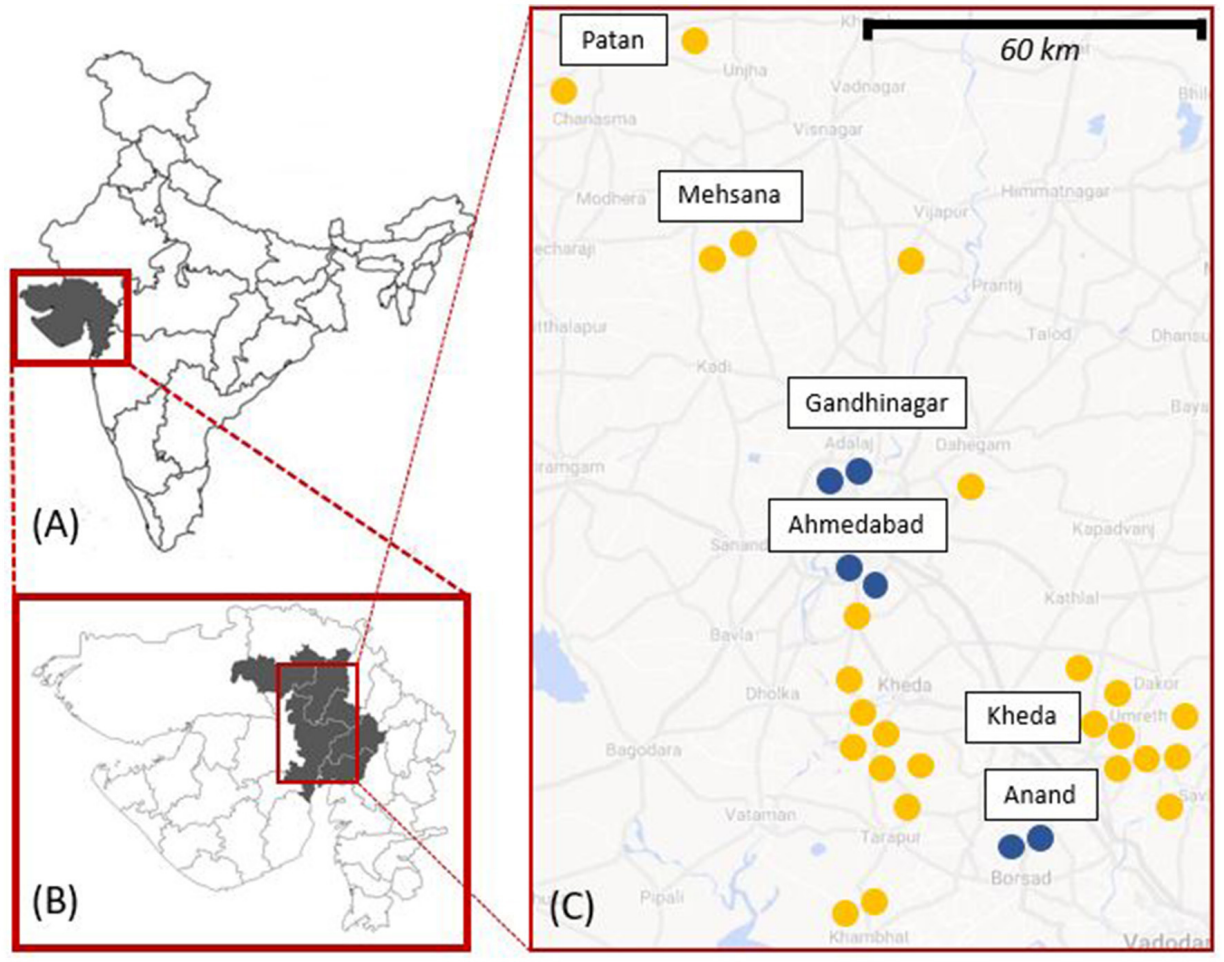
Map showing: **(A)** Gujarat; **(B)** the six districts of Patan, Mehsana, Gandhinagar, Ahmedabad, Kheda, and Anand; and **(C)** the approximate location of the 31 study sites (25 rural, in yellow, and 6 urban, in blue).

Merging information from Google Maps, YouTube videos of religious festivals, and village-by-village exploratory trips undertaken by DN (anthropologist) in September 2019, 139 places of worship were located. Before the COVID-19 pandemic interrupted fieldwork, DN visited 65 of these, selected by convenience and purposive sampling, starting from those in Anand and Ahmedabad districts and the most famous ones in the research area. After excluding little roadside shrines and unattended temples, the remaining 31 places of worship were included in the study (Figure 1) located in six districts of central Gujarat [total population in 2011 = 16,208,494—(35)], mostly in rural settlements.

### Data Collection

In the 31 study sites, DN and her research assistant, RR (a half-Hindu, half-Christian woman born, raised, and college-educated in social work in the city of Anand), met for the first time and interviewed the bhuva (healer) or, when this person was not reachable, the sevak (temple caretaker). The purpose of this research, including the production of scholarly articles, was explained. Participation was entirely voluntary and verbal consent was obtained. On 25 occasions, either one or both among the bhuva and the sevak as well as some co-villagers participated in the discussion. The interviews, lasting 20 to 70 mins, were conducted in Gujarati, in or outside the temple (or, rarely, at the bhuva's home) and were audio-recorded with informed consent. A pre-defined semi-structured interview sheet consisting of 36 open-ended questions guided the conversation, but participants were encouraged to raise unaddressed issues. The first two interviews served as a pilot, but they were exploitable for analysis. All the participants we approached agreed to be interviewed.

Pictures and videos of the temples, Hadkai Mata's sacred images, and ritual paraphernalia were taken, where allowed by temple staff. The research team were often asked to treat the image of the goddess with respect, for example by refraining from printing her pictures to avoid them being accidentally dropped or stepped over. Observation was performed during four healing sessions and the ceremony in which a new bhuva was elected. During and after each temple visit, DN took notes of her own observations, feelings, comments, and thoughts, as well as those of the research assistant and three drivers. The presence of the drivers, all men, proved crucial for facilitating the researchers' approach to those temples where (local) women are not allowed and starting the conversation in vernacular Gujarati.

### Data Analysis

The interviews were verbally translated into English and transcribed by the research assistant, and checked for accuracy by another Gujarati native speaker. Intelligent transcription was chosen to retain substance and accuracy, while ignoring fillers and expressions of agreement (the latter being very frequent, as the answers of the bhuva were rarely contested by the other respondents). Interview transcriptions were then inductively open coded by DN in Nvivo 12, where a double and iterative approach was taken. First, in each transcription, the answer to each of the key questions was identified and coded and the process was repeated for all interviews. Then, all the transcriptions were considered in their entirety and transversal themes were created. Photos, pictures, and field notes were used to enrich the data and referred to for triangulation. All data were pseudo-anonymized for analysis following transcription.

Quantitative data were analyzed in Excel 2019. In the case of multiple respondents and disagreement among them, the opinion of the highest-in-charge person was considered.

Direct quotes, chosen for their representativeness and revealing quality, appear in italics. Data are presented in order of frequency.

## Results

### Characteristics of the Study Participants

The age of the study participants ranged from 25 to 85. All the bhuva inherited their role, which is male-only, from their fathers or older brothers. One election ceremony, observed by DN, where a new bhuva was chosen based on his ability to fall into trance, is a rare event.

All the interviewed bhuva claimed that they provide their service for free, as they usually have another job from which they generate their income, and which allows them to rush to the temple as needed. Elderly bhuva maintained that they are financially supported by their family. While personal donations are officially not accepted, offerings to the temple, both in the form of money (customarily, 5 to 20 rupees, 0.06 to 0.30 US dollars) or food for the deity, are. In the case of healing, donations can be more substantial. The biggest Hadkai Mata temples included in this study are managed by a Trust, which has access to the offerings and decides how to use them. In the smaller temples, this issue was not addressed during the study.

Most of the Hadkai Mata's bhuva and sevak who participated in this study belong to communities historically considered out- (39%) or low- (52%) caste (Scheduled Castes and Other Backward Castes). Almost the totality of the surveyed temples are located in the area of villages designated to these people. Only in a small proportion of the surveyed temples, the bhuva or sevak belonged to a mid (3%) or high (6%) caste. According to the interviewees, high-caste people are rarely involved in Hadkai Mata devotion. The research assistant, drivers, and other Hindu people met during fieldwork confirmed this.

### Reason for Dog Bites and Rabies Infection

When asked the reason for dog bites, 84% of the informants provided an Hadkai Mata-related answer, while the remainder mentioned ethological or biological reasons (for example, part of dog behavior, pups' protection, reaction to danger, and rabies-induced aggression). According to an example often cited by those in the first group, there must be a reason why seven people walk together and a dog comes and bites only the one in the middle. This reason was explained as “*sin*,” “*karma*,” “*being at fault*,” or “*having done something wrong*,” in general or against Hadkai Mata. General mistakes commonly include lying, dishonoring promises, stealing, being stingy, overindulging in alcohol, and disrespecting relatives. The misbehaviors against the goddess mentioned by participants included committing perjury by her name, insulting her or her image, or “*taking her lightly and believing that she has no strength*.” The latter misconduct is considered particularly dangerous, because “*like parents who let go the mistakes of their children once, twice, and then slap them if they repeat it, she* […] *doesn't let go of many mistakes*,” “*she doesn't tolerate anything wrong*,” and “*she doesn't forget and doesn't forgive*.” The most distinctive feature of Hadkai Mata is her strict and resolute nature, to the extent that she is commonly referred to as “*judge*,” “*magistrate*,” “*Supreme Court*,” and “*Nyaya ni Devi*” (literally, the Goddess of Justice). She is famous for being the most severe among the deities that “*when other goddesses have complaints against their* [unruly] *followers, they register a case with her* […] *as we do at the police station*,” and they ask her to “*teach them a lesson*.”

Like most Hindu deities, Hadkai Mata has a companion animal and animal that she rides, which, in this case, is a dog. In the Hadkai Mata-related explanation of rabies, dog bites are the means through which the deity punishes misbehaving people, by infecting them with rabies. She usually gives people a first warning, by sending a “*good* [not rabid] *dog*” to bark at the wrongdoer or nibble on their clothes. If they misunderstand or belittle this signal, she “*sits on a dog*” that, because of the presence of the angry goddess on them, immediately becomes rabid and bites them upon her command. As soon as the bite occurs, “*Mataji's rabies starts*” in the victim. According to one respondent, rabies could start in a person even if the biting dog is not rabid, or without any bite at all. “*If she wants to make a case, her power is enough*.”

Once “*Mataji's rabies starts*,” there are two possible scenarios. People who do not take the goddess seriously or do not care about correcting their mistakes will invariably die. An informant reported of a man that belittled the bite he had received from a pup reckoning that the dog was just playing and not biting upon Hadkai Mata's command. In cases of exceptional devotion, the bitten person may even accept death as the only fair consequence of their misbehavior. This was reported to have been the choice of one bhuva who had operated in one of the surveyed temples until a few years ago. The mistake he ascribed his rabies infection to was kicking a dog, which had eaten his food, while he was on duty at the temple. According to his successor, he refused both the goddess' and doctors' help, asked to be locked up in the temple, developed rabies, and died.

The second scenario applies to those who acknowledge their misdeed and want to fix it. If they follow the necessary healing process, described below, “*Hadkai Mata turns a bad* [rabid] *bite into a normal bite*” and “*takes rabies back*.”

### Healing Process

The healing process as reported by the study participants consists of three consecutive, equally necessary steps.

#### Step 1

**Figure 2:**
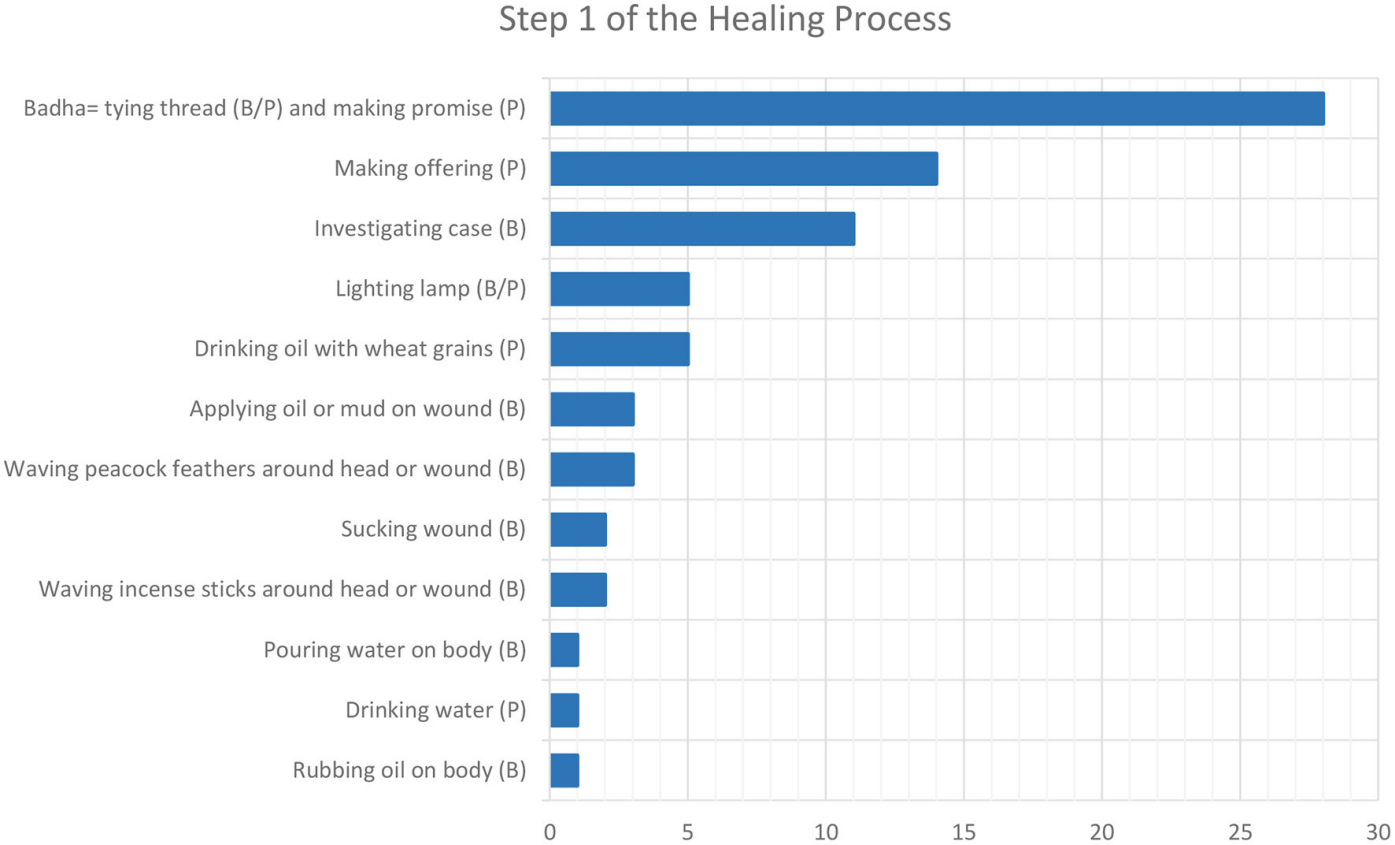
Number of interviewees who mentioned the actions to take (by the bhuva, B, or the patient, P) during the first step of the healing process.

Step 1 usually involves the visit to an Hadkai Mata temple, where at least one of the activities illustrated in Figure 2 is performed. The most important ritual, mentioned in 95% of the interviews, is *badha* (literally, bond, restriction), or tying a thread around the wrist or neck of the patient, while the latter makes their promise to the goddess. The informants speculated that in most cases, the promise involves a future offer to Hadkai Mata in exchange for her doing the requested “*work*” for the follower. Provided that “*the promise is made with your full heart*,” then “*everything* [the person's chances of healing] *is in the thread*,” which purpose is to “*build faith*” and maintain it during Step 2. The wound is treated very rarely, because “*Mataji is the medicine and she is the doctor*,” so “*people apply faith and go*.”

##### Investigation

In one-third (n=11) of the temples, the bhuva runs a test to understand the presented bite case and how to handle it. Ten bhuva use wheat grains and one uses water, which is splashed on the patient's face to check their reaction. A bunch of wheat grains is thrown on a flat surface several times and, depending on the sequence of even and odd results, the answer to the bhuva's query is understood. There are usually three kinds of queries.

The first one concerns the status of the dog and the dangerousness of its bite: “*good dogs*” have a “*sweet bite*” that is usually not a concern, while “*bad dogs*” have a “*bitter bite*” for which the healing process must begin immediately in the hope that “*Hadkai Mata will make it sweet*.” “Good dogs” are usually pet dogs or dogs whose bite was provoked, for example when a person inadvertently steps on their tail. “Bad dogs” are those who show the signs of rabies (described by the interviewees as hypersalivation, aggressiveness, attempts at chewing objects, restlessness, and tail bent between the legs) or unknown dogs that “*act upon Mataji's order*,” so they appear only to bite the designated person and then run away.

The second query regards the mistake that led to the dog bite. Most bhuva leave the patients to determine their misbehavior on their own, but in a few temples, a more public acknowledgment is considered crucial for the recovery process to help the victim be fully aware of their fault.

The third query is about the chances of success of the healing treatment. When the victim shows evident signs of rabies, the bhuva will tell, by reading wheat grains as described above, whether the case can still be solved by Hadkai Mata or if “*it is spoilt*” (or ruined, doomed to fail).

##### Start of the Healing Process

In most cases, the thread is tied by the healer, but in some circumstances, this can also be done at home by the bite victim themselves, if a relative of them goes to the temple and collects the thread. These circumstances include (1) people who live too far from the temple; (2) people who are too worried to wait until going to the temple; (3) women who are menstruating (who would in any case be treated outside the temple, as it happens to any women in those temples where only men and girls are allowed); (4) people who belong to a caste lower than the one that runs the temple (where caste segregation rules are applied); and (5) people whose faith is particularly strong. When the healing process begins at the temple, Tuesdays or Sundays are the best days for two reasons. First, these are the most auspicious days to receive help from Hadkai Mata. Second, while the biggest temples are usually open 7 days a week, minor ones are attended by the bhuva only on these 2 days.

All the informants agreed on the importance of starting the healing process as soon as possible and within 3.5 days in all cases. “*Even if they* [bite victims] *come half an hour* […] *before this time, then she can save them. After that, Mataji says that ‘My time is over, now I cannot do anything. Nor can any doctor do anything’*.”

According to most study participants, 3.5 days is also the time it takes for rabies clinical signs to show. A few mentioned a period of 1, 3.5, or 6 months for rabies signs to become evident, but a bhuva observed that “*she will wait for 2 years and she will show the symptoms. Like if I owe you some money, I will forget, but will you forget?*” Similarly, Hadkai Mata never forgets.

If a person reaches the temple later than 3.5 days since the bite, the healing process can still begin, if Hadkai Mata expresses her approval through the wheat grains, but the bhuva “*cannot give any guarantee*” of success. High diabetes was often taken as an example to explain the difficulty of avoiding death when “*rabies is full*,” and 42% of the informants claimed that there is no chance of survival if the person starts the healing process after the 3.5-day limit. Death is expected to occur within—again−3.5 days since the onset of symptoms, or, if rabid patients are splashed with water or exposed to bright light, right away.

Seven informants reported cases of people that developed rabies symptoms earlier than expected, were rushed to an Hadkai Mata temple within 3.5 days, and were cured as soon as the bhuva asked the goddess for an emergency healing and performed their rituals (i.e., having the patient drink oil or waving peacock feathers around them). Critical cases are often sent to the temple in Kotha that, unlike minor temples that “*are like simple lawyers, where people can only make their promises*,” “*is like a court*,” where final decisions are made. “*People with full rabies*” were described as being tied up because of their agitation, salivating abnormally, scared to drink, bathe, and look at a light, fire, or red objects, restless when air is blown on their face, and unable to stand still.

##### Role of the Bhuva

This section, and any other mention of the role of the bhuva, presents the position of the healers from their own perspective. Their patients may provide a very different portrayal of the bhuva.

Bites from dogs—and dogs only—to people or their livestock are the main reason why people go to Hadkai Mata temples and benefit, if they wish to, from the services of the bhuva. A wide range of other deities are locally available for other needs. However, as with other powerful goddesses, people occasionally address Hadkai Mata also for generic reasons, which do not require the bhuva's assistance, such as having a (male) child, finding a good job, or solving visa-related issues. On Sundays, some major Hadkai Mata temples, such as the one in Patan, often become the informal venue for the members of a specific community, the Devipujaks, who consider Hadkai Mata their tutelary goddess. This is an occasion to spend the day together, celebrate the deity, and discuss, among themselves and with the bhuva, mundane issues such as disputes over money borrowing or extramarital affairs.

In almost all the interviews, the informants specified that the main role of the bhuva is being available as middle-persons that can easily communicate with Hadkai Mata. All but one of the interviewed bhuva strategically claimed that they never present themselves as those who have the power to cure, because that can be done only by the goddess. While this denial of personal responsibility emerged clearly in the interviews, what bhuva say they are happy to do is telling Hadkai Mata something like “‘*Cure everyone, they are all your children. If they have done some sin, slap them and give them some small diseases, but don't give them rabies’*.”

Similarly, they always stressed that the matter is resolved between the goddess and the patient, not the bhuva. Bite victims are encouraged to discuss their situation directly with Hadkai Mata, as a sevak explained: “*I tell them ‘You tell Mataji yourself, this is not my work’. This is the era of science* [compared to the previous generation of bhuva, today's bhuva increasingly deal with patients who have received a formal education]*; we should not fall into this* [today's bhuva should not put themselves in a position that makes them vulnerable to criticism by educated people].” Promises too must be addressed to the goddess, not the bhuva. “*We are only taught that by worshiping her, rabies can be cured. We don't know what the secret behind it is. We have faith in the goddess. We follow moral values, rituals, and tradition*,” a bhuva clarified.

#### Step 2

**Figure 3:**
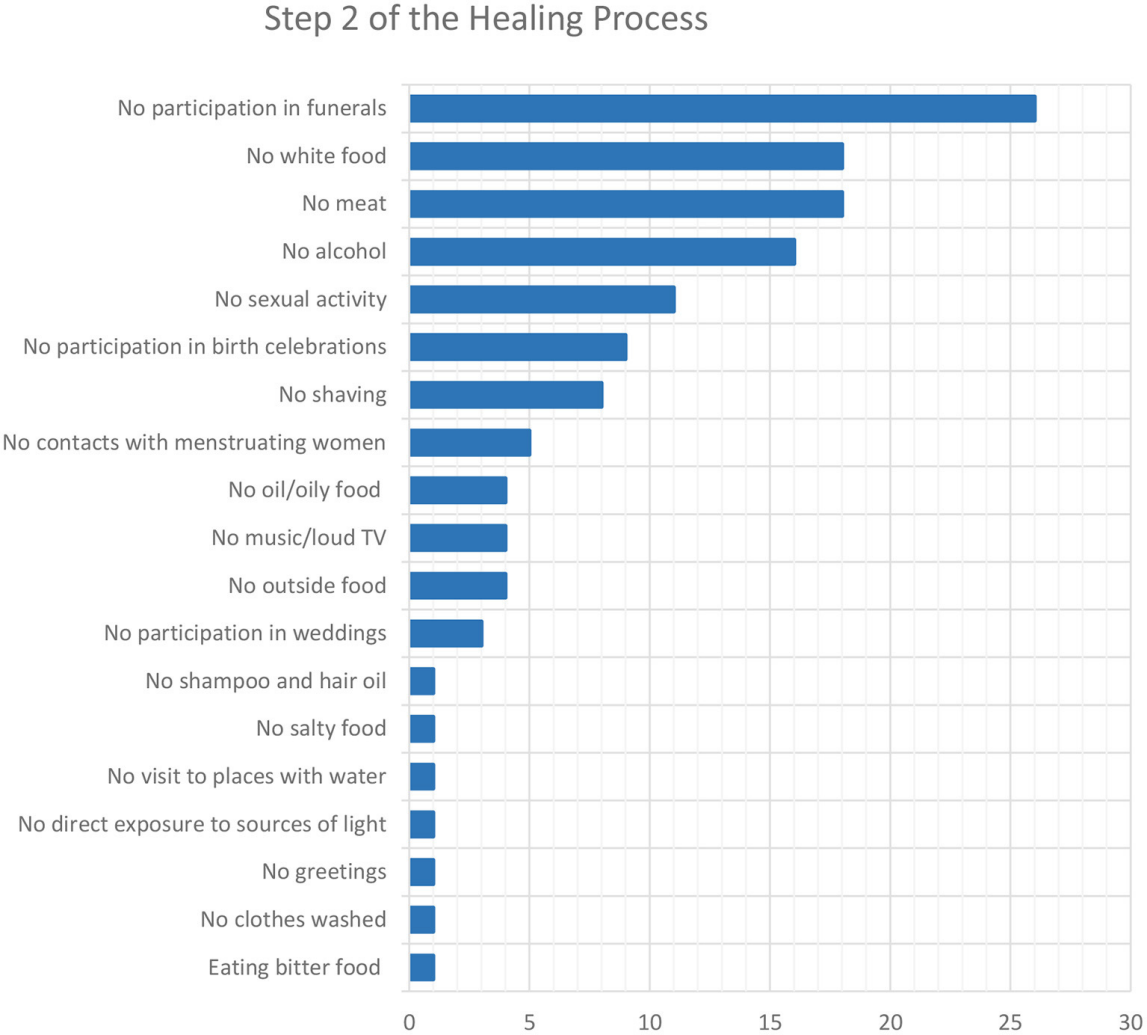
Number of interviewees who mentioned the rules to follow during the second step of the healing process.

From the moment the promise to the goddess is made, patients must abide by precise rules that, according to most informants, are often already known by the patients themselves (Figure 3).

Almost all the study participants agreed that these rules apply for 5 weeks. One respondent claimed that 8 days are enough, while two said it depends on the personal agreement between the patient and Hadkai Mata (for example, when the person has to attend a wedding during the 5 weeks).

Diverging opinions were given with regards to what happens if these rules are broken. Going to funerals or to houses where a child was born, drinking alcohol, or eating meat is particularly risky and an immediate, probably lethal, “*rabies attack*” is likely to happen. According to one bhuva, this fatal event happens even if the person is undergoing PEP but infringes a rule. In particularly stubborn cases, the goddess may have a rabid dog bite not the offender themselves, but a dear family member or their livestock. Even worse, “*Mataji will have such a full power that rabies can be spread from person to person via touching*.” In case of minor offenses, patients “*have to face the anguish of Mataji*” in any case but, upon the payment of a fine to Hadkai Mata (for example, a coconut or a sum of 0.35 or 0.70 USD), no negative consequence will occur. To minimize risk, an informant pleads with people to temporarily free themselves from the promise by untying the thread during the break they need to take. Another said that sometimes bite victims, especially men who travel a lot for their work and cannot always be sure about the food they are offered, have their wives keep the promise on their behalf and follow the rules that this implies.

#### Step 3

At the end of the 5 weeks, bite victims must go back to the temple to make an offering to Hadkai Mata. Sweets are the most common thanksgiving presents, together with dog-shaped toys or metal statuettes. According to four informants, patients should also do things like throwing away the clothes they wore the day they were bitten, lighting a lamp, drinking oil, or undergoing a water test. Clean water is poured into a vessel and shown to the bite victim: if they see “*insect-like things*,” it means that “*they are still at fault as per Mataji*,” so “*she is still in them*.” In this case, the person has to consult any healer in their village to understand the precise mistake the deity is punishing them for and adjust the healing process accordingly.

If a person forgets to go back to the temple, ignoring the dogs that they see in their dreams, where they are sent to by Hadkai Mata as a reminder, they will get punished by being re-infected with rabies, through a second dog bite or simply upon the will of the goddess. No matter what the patient does, this time they will invariably die because the goddess feels she has been cheated.

#### Final Outcome

If people act appropriately during the entire healing process, this typically ends successfully because Hadkai Mata “*always deals with people in accordance to their behavior*.” Patients are considered cured or out of danger when they do not develop any rabies symptoms within 3.5 days since the bite, when their symptoms disappear, or, according to one informant, when they excrete in their feces white tiny *jiv* (literally, germs). In rare cases, the bhuva may suspect that “*the case is still pending*” and recommend that the rules for Step 2 be extended for some months. This precaution is taken especially when Hadkai Mata is punishing the wrongdoer on behalf of another, unsatisfied goddess, who may work on a different schedule.

All the informants stated that Hadkai Mata is always able to “take rabies back” from a person, if she chooses to do so. Nevertheless, fatalities occasionally happen, when (1) people do not have complete faith in her, so “*their mind is not satisfied*”; (2) “*they think that this is all superstition*”; (3) “*they have already sinned a lot*”; and (4) they are too late in seeking her help.

### Opinion About Post-exposure Prophylaxis

The question as to whether PEP should or should not be administered divided respondents. Eight were against this option and tell their patients that there is no need for vaccination, because Hadkai Mata has enough power to cure rabies. According to one study participant, if people go to the hospital, rabies symptoms immediately start because “*the doctor and Mataji are enemies*.” Another said that hospitals and doctors cannot be mentioned in the goddess' presence. “*For fever and other natural diseases, we have to go to the doctor*,” a sevak explained, “*but that is a different thing*.” When the person starts PEP, “*she won't take any responsibility*” and she can deny help. Several informants justified their opinion by observing that doctors also go to the temple in case of a dog bite, and that hospitals often refer their rabies cases to them. They also stressed that, as PEP is free in government hospitals, people must prefer temples over hospitals not because of money issues, but because of their faith.

Eight of the respondents reported believing that PEP can be taken, but only in two situations. The first is that the person goes to the temple first and asks, *via* wheat reading by the bhuva, and obtains permission from Hadkai Mata. The general opinion is that “*if they first climb the steps of the hospital, then nothing will work here* [at the temple],” both because Hadkai Mata would be annoyed and because people would have wasted precious time. Should patients take PEP despite the goddess' negative answer, “*their case will be spoilt for sure*.” That said, the underlying idea that “*Why do we need vaccination when we have Mataji sitting here for us?*” is found in this group too, and is legitimized by the fact that “*here it never happened that someone came and didn't get cured*.” The second circumstance in which PEP can be taken is when symptoms are so severe that Hadkai Mata could struggle to cure the patient.

Seven study participants had a neutral opinion and prefer not to discuss PEP with their patients. Some said that they cannot explicitly recommend PEP, but they are fine if their patients take it—especially the doubtful ones and those who belong to communities that are not particularly devout to Hadkai Mata. This also reflects the fact that they do not want to be blamed in the future for advising them against vaccination. That said, most of these informants personally believe that Hadkai Mata's help is sufficient.

Eight informants considered PEP necessary and claimed that they usually recommend taking it, specifically within 24 h. Even if “*Mataji says that if you have any doubt, then you take vaccination*,” according to these informants “*95% of people don't need vaccination because they have faith in Mataji*.”

Three informants spontaneously mentioned the number of injections that, to their knowledge, are administered for PEP; 5, 14, and 20 injections.

### Management of Rabid Dogs

“Bad,” rabid dogs are always described as free-roaming and mostly unowned. Unlike people and livestock, Hadkai Mata cannot cure them because “*no one would go that far for a street dog*,” by trying to catch them and committing to a 5-week obligation on their behalf. Hence, rabid dogs invariably die—again—after 3.5 days from the moment they were ridden by the goddess and became rabid, or as soon as somebody throws water on them. Pet dogs could potentially be cured, but it is very unlikely for them to become rabid, because Hadkai Mata only selects dogs that can move freely and walk long distances—“*even 100 km*”—to bite the targeted person. Only two respondents said that rabid dogs can be cured, either by giving them buttermilk to drink or when they spontaneously perform pradakshina (i.e., the ritual circumambulation of an Hadkai Mata temple).

Diverging approaches to rabid dogs were found. Half of the respondents said that dogs are killed, both to keep people safe and to avoid, especially during the wedding and festival seasons, that bite victims are bound to Step 2-rules and cannot enjoy social events. The common belief that Hadkai Mata would not cure her dogs anyway, because of the lack of a human intermediary, was often provided as a justification for dog killing. In two temples, food is offered to Hadkai Mata as a self-imposed community fine when a dog is killed.

The other half of the interviewees claimed that rabid dogs cannot be killed, because the goddess is sitting on them and she cannot either be hurt or disrespected by killing her companion animals. A bhuva tells his co-villagers “*to shut their doors and* […] *wait for the dog to pray at the temple* [when they finish the job of biting people assigned to them by Hadkai Mata]” and leave the village for good. In one village, food is offered to the animal as a ritual before their departure.

### Opinion About Mass Dog Vaccination

All the 11 informants with whom mass dog vaccination was discussed hypothetically said they dislike this measure. The objection is that dog bites are useful to and wished by Hadkai Mata and the other goddesses that request her assistance to discipline their followers. Mass dog vaccination is considered a risky interference in Hadkai Mata's work, from which “*it is always good to stay away*,” because of the power of the goddess. One bhuva made his position clear by saying that “*rabies is not a disease, it's the wish of Mataji*,” so he would not support anything that goes against her will.

## Discussion

For many diseases, endemic countries have developed specific policies, often supported by the WHO Traditional Medicine Strategy (45), to strengthen the role traditional medicine plays in keeping populations healthy. Because of the clinical incurability of rabies, no such policy exists for this disease. Yet, work from Vietnam shows that traditional healers can be successfully engaged in rabies control programs to better connect with local communities and increase the chances that bite victims, especially in rural areas, receive precise information about PEP from a person they trust (46). Even though faith healers can be harder to involve than other traditional healers (47), encouraging examples can be found in the field of mental illness where healers and psychiatrists work as a team. For example, in central Gujarat, the Department of Health and Family Welfare has established the collaborative project “Dava & Dua” (literally, medicines and prayers) at a famous Muslim shrine (48).

In the context of devotion to Hadkai Mata, many key points of divergence—but also some of convergence—with the technical solutions of the NAPRE can be observed and, hopefully, discussed and worked upon. The participants in this study, including those who do not believe in vaccination, never expressed feelings of hostility or resentment toward biomedicine or doctors (for example, rumors about PEP and stories of disservices). However, this could be due to the lack of confidence in expressing this view to an unfamiliar research team. Nevertheless, they were proud of having—according to them—doctors among their patients, and cases referred from hospitals. Incidentally, the first time DN heard about Hadkai Mata was in a newspaper article that mentioned the presence of the goddess' portrait in a rabies clinic in Ahmedabad. Additionally, at least in minor temples, bhuva are not materially compensated for their service (even though they benefit from high social recognition) and this presumably reduces the risk of non-cooperation because these people's source of income lies elsewhere. That said, the fact that one quarter of the interviewed bhuva are opposed to PEP, and only another quarter explicitly recommend it, is worrisome and calls for dialogue and engagement with local faith healers. The success of this dialogue depends on the external interlocutors keeping in mind that Hadkai Mata controls not only rabies but, through it, social ill-being. Looking back at the extended parallel processing model presented at the beginning of this paper (according to which fear and self-efficacy are the main drivers for effective health-seeking behaviors), in this context, the factor of fear is anything but straightforward. Anti-social behaviors are feared as much as, if not more than, rabid bites, so these two issues need to be discussed and addressed as a unit.

According to the complex explanatory model of rabies that we started to explore in this study, this disease is considered supernatural and triggered by a person's misbehavior (usually in the domain of social relations) that forces Hadkai Mata to take the action that is expected from her. This is particularly evident in the belief (or at least in the profitable recommendation, from the perspective of bhuva) that if a person forgets to go back to the temple at the end of their healing process to duly thank Hadkai Mata, the will of the enraged goddess is able—alone, with no dog bite—to fatally re-infect the unruly patient. This etiological explanation is unusual, as causes of disease are usually sought, and found, *outside* the victim and their responsibility [for example, evil eye, witchcraft, immigrants, poor—(49)]. Nevertheless, those informants who provided ethological and biological explanations for dog bites and the frequent mention of germs in the wound suggest the co-presence, in this healthworld, of a vague, distant, underlying biological source of disease (i.e., the rabies virus), and a much more concrete, close, immediate cause of illness (i.e., Hadkai Mata's punishment). This double interpretation has been observed in other contexts of medical pluralism (50). Rabies as an illness, perceived as a priority for the psychological and social repercussions of misconduct, is treated at the temple. There, the patient and the healer share the same conceptualization of ill health and the same socio-cultural background and related challenges (for example, caste-based ostracization), and almost wordlessly work toward the same goal of moral refurbishment. Rabies as a disease can, meanwhile, be managed—just in case—via PEP, as most bhuva and sevak do not see vaccination and Hadkai Mata's healing powers as incompatible.

In our study, the idea that “*Hadkai Mata is for 3.5 days*” is central. The 3.5-day limit for seeking treatment leads to two observations. First, the belief that healthcare should be sought first and foremost at the temple, preferably on Tuesdays or Sundays, dangerously delays PEP (for those who choose it). In India and abroad, traditional medicine has already been found to delay vaccination (51). This is an important concern, because wound washing is rarely performed (52, 53) and timely access to PEP is already delayed for a wide array of reasons, such as, according to a study done in Baroda, Gujarat, work pressure (especially for daily laborers that do not have a fixed salary), forgetfulness, low bite severity, travel, and financial constraints (54). The fact that treatment at temples takes only 15 mins, there is minimal to no waiting time, bhuva are always available on Sundays and usually easy to source during the rest of the week, and family pressure is high makes faith healing more easily and quickly accessible than hospitals. At least in (urban) areas where anti-rabies clinics are open 24 × 7, information about the need for immediate PEP and the opening hours of clinics should be widely circulated. Importantly, other factors that undermine the access to healthcare by socially marginalized communities, such as caste-based discrimination by healthcare providers, need to be structurally addressed (55).

The second observation is that, despite the popularity of treatment at temples, the urgent need for PEP in case of a dog bite was also recognized by some respondents. This provides a solid starting point for consolidating and expanding access to vaccination. Unlike in other parts of India (56), most hospital-based studies from Gujarat found that the majority of patients sought medical care within 24 h, or 48 at the latest, of the bite (57), probably because of sufficient road connections and widespread health infrastructure. In India, government hospitals are preferred to private ones by dog bite victims (52) and in our study area, local Primary Health Centres (58) are preferred. In these facilities, it is therefore essential to ensure support for effective delivery of PEP, including the continuous availability of vaccine and RIG stock, staff expertise (for example, especially on intra-dermal vaccination and RIG administration), wound washing facilities, convenient opening hours, and, more importantly, a cost-free service. When fees are charged (43), economically marginalized communities are disproportionately affected, and this may increase the preference for traditional and faith healing. At most of the visited Hadkai Mata temples, donations are usually discretionary. Furthermore, no more than two temple visits are necessary, while at least three hospital visits are necessary for PEP, increasing travel-related costs.

Given that the onset of clinical signs and symptoms is not a central crux in the Hadkai Mata-related exploratory model of illness, awareness messages should stress the fact that PEP can *prevent* this from happening. Particular attention has to be paid to the translation, across conceptual and linguistic healthworlds, of “preventable,” “treatable,” and “curable.” Messages should also be clear about the necessary number and prescribed site of PEP injections. Even though the use of Nerve Tissue Vaccines was discontinued in 2004, its painful and numerous injections in the abdomen still persist in the memory of adults and the elderly in many parts of India (59), and probably informed the belief that dog bite victims become pregnant with the puppies of the biting dog (60). The prospect of 10 to 14 injections during as many hospital visits (58) is evidently discouraging for impoverished bite victims, if only for its devastating impact on income and travel-related expenditure. In a survey in rural Anand district, hence in the same area of our study, one-third of the respondents believed that rabies is curable, and mentioned tetanus injections as a first-aid measure. In the city of Ahmedabad, the biggest city in Gujarat, 61% of interviewees are not aware of the fatality of rabies and 55% would not get PEP if they do not have any symptoms (61). Increasing awareness about the correct, short, and pain-free modern PEP calendar may increase compliance after the initial PEP doses, which is a problem both in India (57) and abroad (62). This information should be shared in a detailed yet easily understandable format that takes into account the different levels of literacy and biomedical knowledge in the population (for example, by clearly distinguishing life-saving PEP from tetanus vaccination, which can also be essential). A similar “awareness update” on wound washing and PEP technicalities should target also healthcare providers, whose practical, first-aid knowledge is not always adequate in endemic countries (63). As one sevak observed, “*this is the era of science*” so, as is already the case abroad (64) and in some Indian States (65), simple but practical information should be spread to all school children, as a long-term investment in their health and that of their communities.

In our study, the main point of divergence is dog vaccination. Resistance can be due to the local interpretation of rabies transmission, in which dog-to-dog transmission receives little attention, but also to the inexperience of rural residents in this matter. Dog rabies vaccination campaigns are rare in rural India, and among dog-related issues, rabies is a low-priority concern for Indians (66). A study in Anand, in our fieldwork area, found that only 6% of respondents believe that dog vaccination controls human rabies, while 76% did not consider rabies vaccination important in pet dogs (58). In rural India, veterinary consultation for dogs is low (10), arguably due to both the negligible economic value of dogs compared to livestock and also the generally low position of (free-roaming) dogs in Hinduism (67) and Islam (68). Structural drivers such as poverty and limited interest in dog health from the government veterinary sector contribute to poor dog welfare standards. We, therefore, make three recommendations. First, even though some respondents in our study mentioned dog-to-dog transmission of rabies, human and dog rabies are deeply interlinked in the Hadkai Mata-related healthworld. Hence, an internally congruent and synchronized One Health strategy is fundamental. For example, it could be emphasized that dog vaccination, while preventing a dog from becoming infected by rabies, does not alter dog behavior [as feared in South Africa—(69)], or automatically reduce dog bites (which may already be commonly experienced from healthy dogs). In the beginning, insisting on dog bite prevention could be counterproductive, because of the value assigned to biting dogs as a means to social health. Second, given that in rural India pet dogs are usually free-roaming, the message should be conveyed—in a culturally-sensitive way—that these dogs can also be rabid. Third, instead of focusing on individual dog ownership practices, it seems more sensitive and effective to work on community-based dog keeping practices and values (as demonstrated, for example, by the strong social cohesion and shared responsibility that emerge when villagers agree with not killing rabid dogs and waiting for them to leave). Considerable work will be needed to develop strategies for controlling rabies through dog vaccination [including oral vaccination approaches—(70)] that can be implemented effectively and acceptably in these communities.

### Strengths and Limitations

This anthropological paper is the first on Hadkai Mata, probably the only goddess, in any current world religion, worshiped specifically in case of rabies exposure. We provided rich and from-the-ground-up data on the Hadkai Mata-related healthworld with regards to both human and dog rabies. Having all the interviews carried out by the same research team allowed for consistency in data collection. For many of the discussed topics, saturation was reached and courtesy bias seemed minimal. Nevertheless, because of the pandemic, we could not discuss our results with the study participants and collect more interviews. More importantly, we could not perform the planned contact tracing interviews starting from Hadkai Mata temples, to retrospectively chart the patients' journeys through faith-based and, probably, biomedical healthcare; document their actual actions and underlying reasons (research question 2); collect their views on dog keeping and dog vaccination; and—above all—understand the structural challenges they face as socially and economically marginalized people in accessing livelihood assets and, when exposed to rabies, healthcare (71). This contact tracing study would have provided us with quantitative information about the extent to which the bhuva's recommendations are actually followed by dog bite victims. From a qualitative point of view, as most of the dog bite victims that seek Hadkai Mata's protection are likely to belong to the same social group of most bhuva—low-income, low-caste, poorly educated—we do not expect major discrepancies in how Hadkai Mata and her management of rabies are imagined. Nevertheless, the perspective of temple goers is necessary to complete the description of the bhuva and their role in attaining physical and social health. Coding was performed only by one researcher (with a background in South Asian studies), but problematic passages were discussed with the research assistant. As this study refers to a particular social and religious context, it may have low external generalizability. However, traditional and faith healing for rabies likely exists in all endemic countries. The approach we used to explore this issue can be applied to all healthworlds, of rabies and any other disease.

## Conclusions

This study approached the system of knowledge of rabies collectively built by faith healers around the goddess Hadkai Mata in rural central Gujarat through the concept of “healthworld,” an important analytical tool for the field of health in its social context and a perspective toward decolonizing research and public health agendas (72). In the social and religious setting under study, dog bites are both feared and valued, demonstrating once more the multidimensionality of the human-dog relationship (59), and the possibility that, from the point of view of One Health, animals could be “more-than-animals” (for example, emissaries of deities). From a practical point of view, the sustainability of rabies control programs in contexts of medical pluralism can be enhanced with: a better understanding of interventions from the perspective of the multiple actors who are involved on the ground, rather than narrowly emphasizing the efficiency of technical solutions; consideration of the social dynamics, norms, values, needs, and inequities; work with—not only for—communities; and starting from points of convergence and enabling factors in order to transform alternative conceptions of healing from obstacles “into opportunities for understanding the worlds of health in which people live, move and have their being” (24).

## Data Availability Statement

The raw data supporting the conclusions of this article will be made available by the authors, without undue reservation.

## Ethics Statement

Ethical approval was obtained from the University of Washington (STUDY00006267)—with reliance approved by the University of Glasgow—the Ashoka Trust for Research in Ecology and the Environment (IRB/CBC/0005/ATV/07/2019), and the Indian Health Ministry's Screening Committee (2019/7418). Written informed consent for participation was not required for this study in accordance with the national legislation and the institutional requirements.

## Author Contributions

SC, KH, DN, and TL: conceptualization and funding acquisition. DN: data curation, visualization, writing (original draft), and methodology. DN and RR: investigation. SC, KH, TL, DN, and ATV: project administration. SC, KH, and TL: supervision. SC, KH, TL, RR, and ATV: writing (review & editing). All authors read and approved the final manuscript.

## Funding

This work received funding from the European Union's Horizon 2020 research and innovation program under a Marie Skłodowska-Curie Grant (751267) to DN, a Wellcome Grant (207569/Z/17/Z) to KH, and a DBT/Wellcome Trust India Alliance Grant (IA/CPHI/15/1/502028) to AV.

## Conflict of Interest

The authors declare that the research was conducted in the absence of any commercial or financial relationships that could be construed as a potential conflict of interest. The reviewer KT declared a shared consortium with one of the authors SC.

## Publisher's Note

All claims expressed in this article are solely those of the authors and do not necessarily represent those of their affiliated organizations, or those of the publisher, the editors and the reviewers. Any product that may be evaluated in this article, or claim that may be made by its manufacturer, is not guaranteed or endorsed by the publisher.
